# Hydroxyl Group and Vasorelaxant Effects of Perillyl Alcohol, Carveol, Limonene on Aorta Smooth Muscle of Rats

**DOI:** 10.3390/molecules23061430

**Published:** 2018-06-13

**Authors:** Ana Carolina Cardoso-Teixeira, Francisco Walber Ferreira-da-Silva, Dieniffer Peixoto-Neves, Klausen Oliveira-Abreu, Átila Pereira-Gonçalves, Andrelina Noronha Coelho-de-Souza, José Henrique Leal-Cardoso

**Affiliations:** 1 Superior Institute of Biomedical Sciences, State University of Ceará, Fortaleza-CE 60741-903, Brazil; carol.cardoso@uece.br (A.C.C.-T.); klausen.abreu@aluno.uece.br (K.O.-A.); atila.pereira@aluno.uece.br (Á.P.-G.); andrelinanoronha@gmail.com (A.N.C.-d.-S.); 2 Civil Engineering Department, State University of Vale do Acaraú, Sobral-CE 62042-280, Brazil; walber_ferreira@uvanet.br; 3 Department of Physiology, University of Tennessee Health Science Center, Memphis, TN 38163, USA; dneves@uthsc.edu

**Keywords:** structure-activity relationship, limonene, carveol, perillyl alcohol, vascular smooth muscle, vasorelaxant effect

## Abstract

The present study used isometric tension recording to investigate the vasorelaxant effect of limonene (LM), carveol (CV), and perillyl alcohol (POH) on contractility parameters of the rat aorta, focusing in particular on the structure-activity relationship. LM, CV, and POH showed a reversible inhibitory effect on the contraction induced by electromechanical and pharmacomechanical coupling. In the case of LM, but not CV and POH, this effect was influenced by preservation of the endothelium. POH and CV but not LM exhibited greater pharmacological potency on BayK-8644-induced contraction and on electromechanical coupling than on pharmacomechanical coupling. In endothelium-denuded preparations, the order of pharmacological potency on electrochemical coupling was LM < CV < POH. These compounds inhibited also, with grossly similar pharmacological potency, the contraction induced by phorbol ester dibutyrate. The present results suggest that LM, CV and POH induced relaxant effect on vascular smooth muscle by means of different mechanisms likely to include inhibition of PKC and IP3 pathway. For CV and POH, hydroxylated compounds, it was in electromechanical coupling that the greater pharmacological potency was observed, thus suggesting a relative specificity for a mechanism likely to be important in electromechanical coupling, for example, blockade of voltage-dependent calcium channel.

## 1. Introduction

Monoterpenes and monoterpenoids, the major chemical components of essential oils, are made of two isoprene units [1,2]. They have shown several pharmacological activities such as antioxidant, anti-inflammatory, antiedematogenic, gastroprotective, and anti-hypertensive [3,4,5,6,7]. In addition, monoterpenes have been reported to exert significant effects on the cardiovascular system [8]. Carvacrol, thymol, eugenol, methyleugenol, 1,8-cineole, and terpinen-4-ol are small monoterpenoids, which exhibited vasorelaxant effects on isolated rat aorta [9,10,11,12,13,14]. Moreover, 1,8-cineole and citronellol have been reported to elicit hypotension in normotensive rats [15,16].

Several studies have investigated the correlation between the chemical structure of monoterpenes and their biological/pharmacological activity. For example, the presence of oxygen on the monoterpenoid molecule contributes to the relaxant activity of rotundifolone on both ileum and mesenteric artery [17,18]. Additionally, the position of ketone and epoxide groups on terpenes and terpenoids influences their vasorelaxant activity on rat mesenteric arteries [17]. Previous studies also demonstrated the importance of the hydroxyl group (–OH) of terpenoids in blocking the compound action potential of sciatic nerves and on ionic channel activity [19,20,21,22]. Accordingly, the presence and position of functional groups such as the hydroxyl group, may influence the pharmacological potency and efficacy of these molecules.

**Figure 1:**
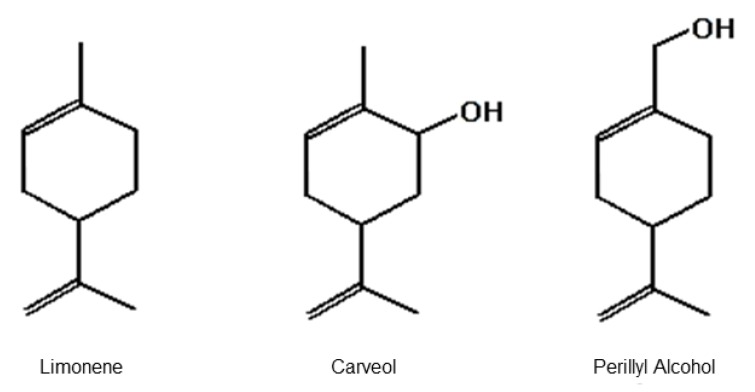
Chemical structure of the monoterpene limonene and monoterpenoids carveol and perillyl alcohol [8].

Three molecules are particularly suitable to evaluate the activity of the hydroxyl group on contractile properties of vascular smooth muscle (VSM): limonene (LM), carveol (CV), and perillyl alcohol (POH) (Figure 1). These terpenoid molecules share a similar structure; however, LM does not have the –OH group, POH is a primary alcohol, and CV is a secondary alcohol [23]. LM, a monocyclic monoterpene constituent of several citrus essential oils, has antinociceptive, gastroprotective, antitumor, and anti-inflammatory properties [24,25,26,27]. CV and POH, hydroxylated metabolites of LM, have repellent and anticarcinogenic properties [2,28,29]. Regarding cardiovascular activities, LM has been shown to relax the superior mesenteric artery in rats and POH has been reported to have a hypotensive effect and relaxant activity on the coronary artery in pigs [17,30,31]. The vasorelaxant effect of CV on smooth muscle preparations has not been described yet. As LM and POH are known to exert pharmacological activities on VSM and CV is likely to exert it due to molecular similarity to LM and POH, the present study compared the acute vascular effects of LM, CV, and POH on the rat aorta. We also studied the possible implications stemming from the presence/absence of the hydroxyl group on this vasorelaxant effect.

## 2. Results

### 2.1. Effect of Limonene, Carveol, and Perillyl Alcohol on the Resting Tonus of Rat Aortic Rings

**Figure 2:**
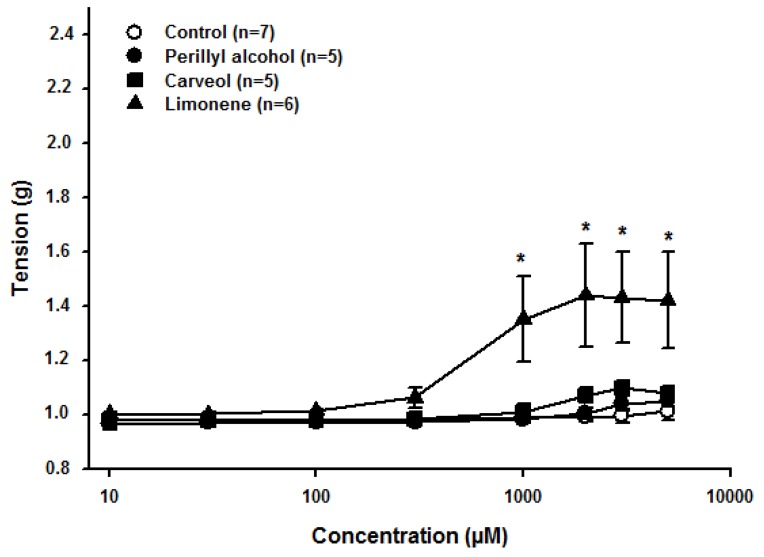
Effects of limonene, carveol, and perillyl alcohol on the resting tonus of rat aortic rings. * indicates statistical difference compared to the control (*p* < 0.05, one-way ANOVA and Holm-Sidak test). Each control consisted of the same concentration of vehicle as in the experimental case, but without the experimental substance.

First, the effect of LM, CV, and POH was investigated on the aorta resting tone. Cumulative concentrations (1–5000 μM) of CV or POH did not significantly alter the resting tone when compared to the control (*n* = 5, *p* > 0.05, ANOVA) in quiescent endothelium-intact aortic rings (Figure 2). In contrast, LM caused a significant increase of resting tone (*n* = 6; *p* < 0.05, one-way ANOVA, followed by Holm-Sidak test) at a concentration of 1000 (0.99 ± 0.01 g in control vs. 1.35 ± 0.15 g, following LM exposure), 2000 (0.99 ± 0.01 g, vs. 1.43 ± 0.18 g), 3000 (0.99 ± 0.02 g, vs. 1.43 ± 0.16 g), and 5000 μM (1.01 ± 0.03 g, vs. 1.42 ± 0.17 g). The vehicle had no significant effect on the resting tone of aortic rings (Figure 2).

### 2.2. Relaxant Effect of Limonene, Carveol, and Perillyl Alcohol on the Sustained Contraction Induced by KCl in Rat Aortic Rings

**Figure 3:**
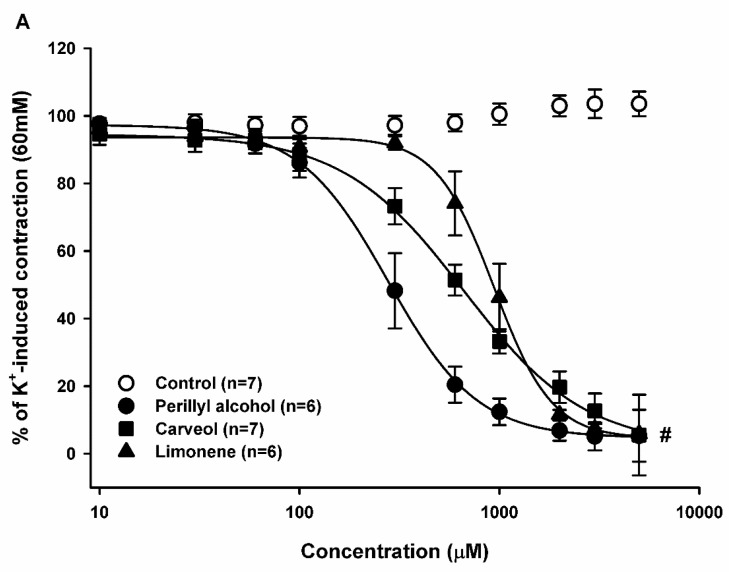

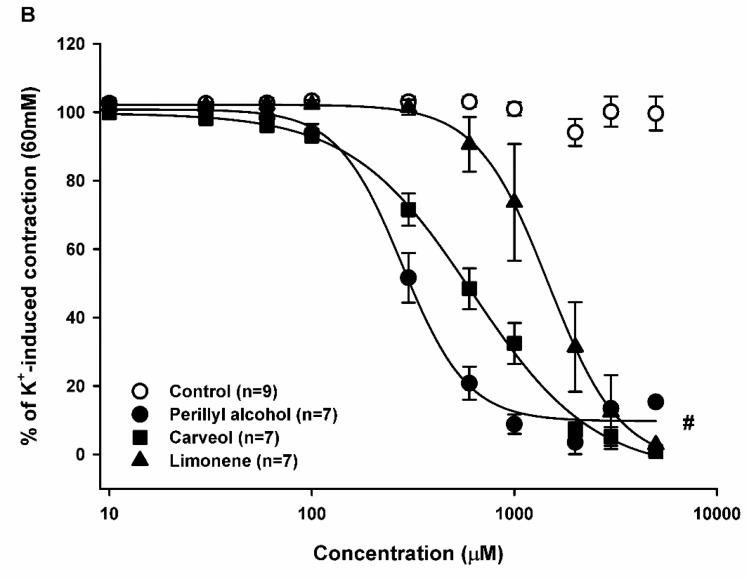
Relaxant effect of limonene, carveol, and perillyl alcohol on electromechanical coupling. Sustained contractions induced by K^+^ (60 mM) in endothelium-intact (**A**) and endothelium-denuded (**B**) rat aortic rings. Results are shown as means ± SEM. # indicates statistical difference of limonene, carveol, and perillyl alcohol compared to the control (*p* < 0.05, two-way ANOVA and Holm-Sidak test). Each control consisted of the same concentration of vehicle as in the experimental case, but without the experimental substance.

To investigate the effect of monoterpene and monoterpenoids on electromechanical coupling (EMC), increasing concentrations (10–5000 μM) of LM, CV, and POH were added to the organ chamber during the plateau of KCl-induced contraction. Addition of LM (*n* = 6), CV (*n* = 7), and POH (*n* = 6) induced a significant concentration-dependent relaxation (*p* < 0.05, two-way ANOVA followed by Holm-Sidak test) on endothelium-intact arteries, with EC_50_ values (concentration at which 50% of the maximal response was observed) of 941.6, 662.1, and 277.7 μM respectively (Figure 3A and Table 1). Endothelium denudation significantly attenuated relaxation induced by LM but not that stimulated by POH and CV. 

In endothelium-denuded arteries, LM (*n* = 7), CV (*n* = 7), and POH (*n* = 7) produced significant vascular relaxation (*p* < 0.05, two-way ANOVA followed by Holm-Sidak test), with EC_50_ values of 1474.5, 619.8, and 279.7 μM, respectively (Figure 3B and Table 1). The relaxations caused by LM, CV, and POH were reversible after washing (data not shown).

### 2.3. Relaxant Effect of Limonene, Carveol, and Perillyl Alcohol on the Sustained Contraction Induced by Phenylephrine (PHE) in Rat Aortic Rings

**Figure 4:**
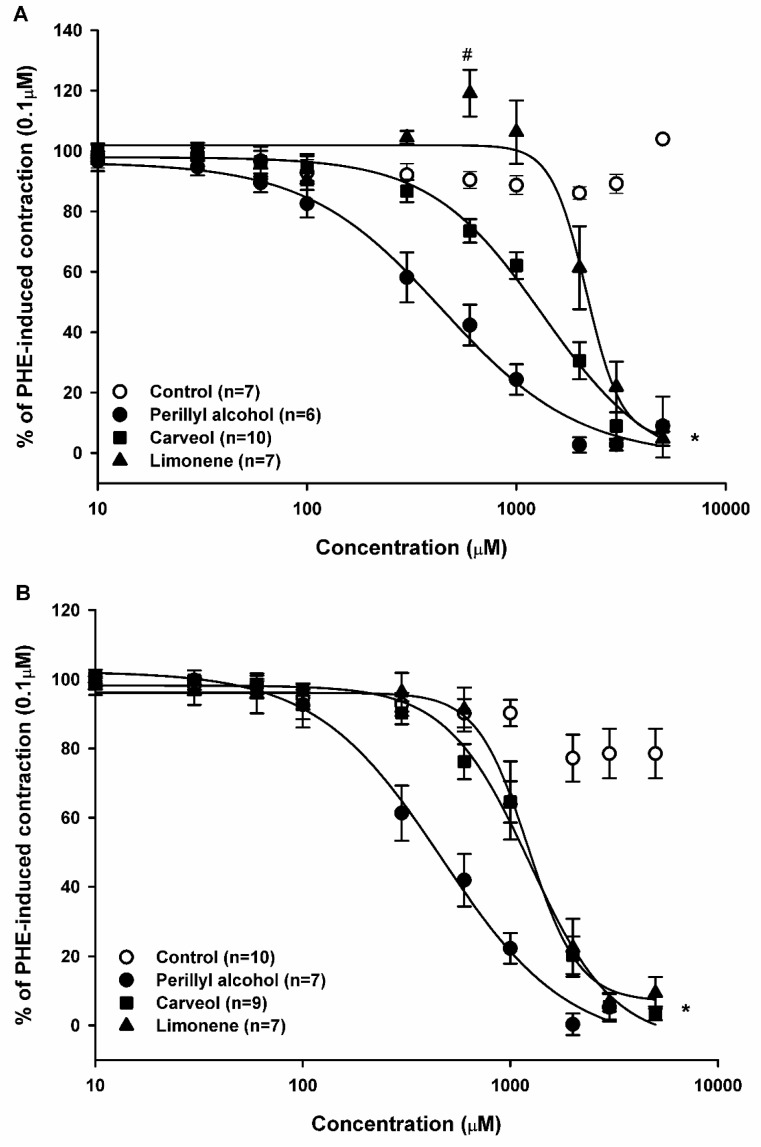
Relaxant effect of limonene, carveol, and perillyl alcohol on pharmacomechanical coupling. Sustained contractions induced by PHE (0.1 μM) in endothelium-intact (**A**) and endothelium-denuded (**B**) rat aortic rings. Results are shown as means ± SEM. * indicates statistical difference of limonene, carveol, and perillyl alcohol compared to the control (*p* < 0.05, two way ANOVA and Holm-Sidak test). # indicates statistical difference between limonene (600 μM) compared to the control (*p* < 0.05, one-way ANOVA and Holm-Sidak test). Each control consisted of the same concentration of vehicle as in the experimental case, but without the experimental substance.

The effect of monoterpenes and monoterpenoids on pharmacomechanical coupling (PMC) was investigated in aortic preparations pre-contracted with PHE (0.1 μM). In endothelium-containing tissue, LM (*n* = 7) induced two distinct responses: a contraction in the 300–1000 μM range (with a significant peak at 600 μM), and a concentration-dependent relaxation at higher amounts. The latter was significant at concentrations above 2000 μM. As seen in Figure 4A, CV (*n* = 10) and POH (*n* = 6) did not exert a contraction but solely induced a concentration-dependent relaxation. Calculated EC_50_ values were 2159.1, 1333.3, and 443.3 μM for LM, CV, and POH, respectively (Figure 4A, Table 1). 

Endothelium denudation significantly altered LM-induced contraction and relaxation, but not CV- and POH-induced effects. In endothelium-denuded arteries, the relaxing effect of LM (*n* = 7), CV (*n* = 9), and POH (*n* = 7) was statistically significant (*p* < 0.05, two-way ANOVA followed by Holm-Sidak test) with EC_50_ values of 1216.7, 1237.3, and 433.3 μM for LM, CV, and POH, respectively (Figure 4B, Table 1).

### 2.4. Relaxant Effect of Perillyl Alcohol, Carveol, and Limonene on Sustained Contractions Induced by BayK-8644

**Figure 5:**
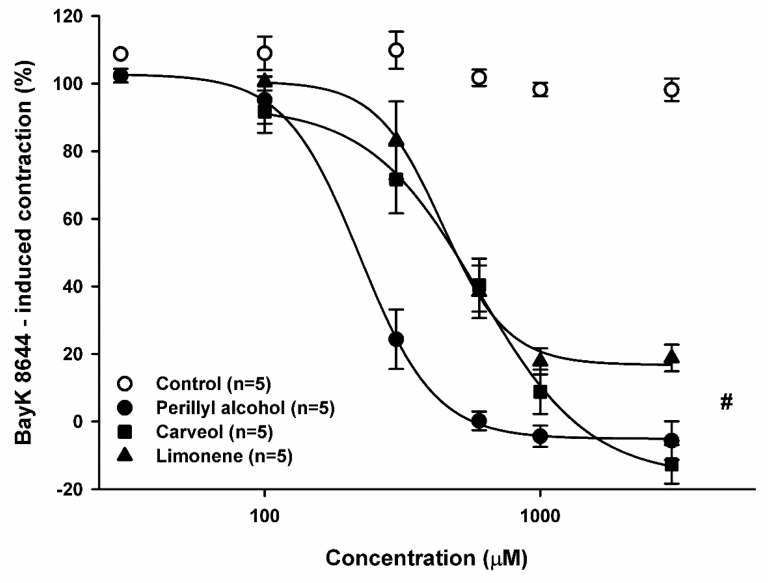
Relaxant Effect of perillyl alcohol, carveol, and limonene on sustained contractions induced by BayK-8644 (2 μM) in endothelium-denuded rat aortic rings. Results are shown as means ± SEM. # indicates statistical difference between limonene, carveol, and perillyl alcohol vs. control (*p* < 0.05, two-way ANOVA and Holm-Sidak test). Each control consisted of the same concentration of vehicle as in the experimental case, but without the experimental substance.

The effect of monoterpenes on contractions induced by BayK-8644, an L-type Ca^2+^ channel activator, was investigated. POH (30–3000 μM), CV, and LM (both 100–3000 μM) relaxed, in a concentration-dependent manner, the contractions of endothelium-containing aortic preparations induced by BayK-8644 (2 μM), and then sensitized with [K^+^] (10 mM) and maintained in Ca^2+^-containing medium. The relaxant effect of POH (*n* = 5) and CV (*n* = 5) was statistically significant compared to the control (Figure 5; *p* < 0.05, two-way ANOVA followed by Holm-Sidak test) with EC_50_ values of 221.4 and 598.2 μM, respectively. These values were similar to those obtained for the vasorelaxant effects of POH and CV on contractions induced by 60 mM [K^+^]. For LM (*n* = 5), the relaxant effect was statistically significant compared to the control (Figure 5; *p* < 0.05, two-way ANOVA followed by Holm-Sidak test) with an EC_50_ value of 439.0 μM. However, LM did not cause full relaxation of the aortic preparation in the concentration range used.

### 2.5. Effect of Limonene, Carveol, and Perillyl Alcohol on the Sustained Contraction Induced by Phorbol 12,13-Dibutyrate (PDB) in Rat Aortic Rings

**Figure 6:**
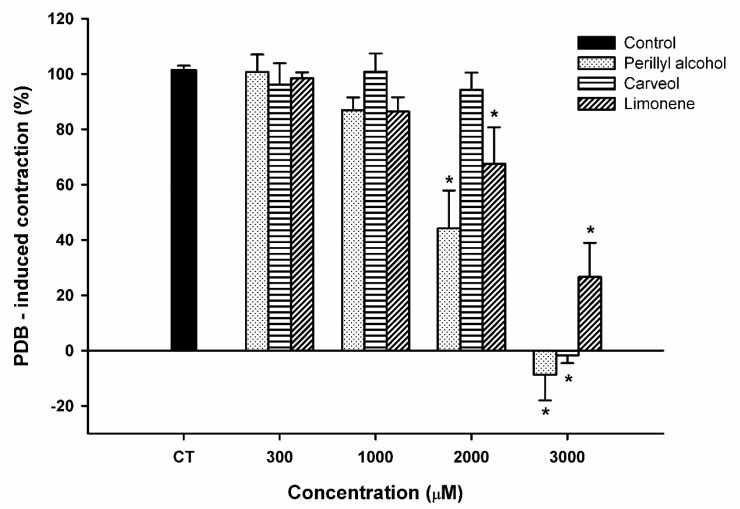
Relaxant effect of perillyl alcohol, carveol, and limonene on sustained contractions induced by PDB (1 μM) in endothelium-denuded rat aortic rings. Results are shown as means ± SEM. * indicates a significant effect (*p* < 0.05, ANOVA on Rank’s followed by Dunn’s test).

To investigate the effect of LM and its analogs in Ca^2+^ sensitization, we used PDB (1 μM), a protein kinase C (PKC) activator [32]. In endothelium-denuded aortic rings incubated in Ca^2+^-free medium and in the presence of thapsigargin (1 μM), a calcium pump inhibitor [33], PDB induced slow and sustained contractions with a mean tension of 1.39 ± 0.08 g. LM and POH significantly reduced PDB-induced contraction by 42.63 and 55.75%, respectively, at 2000 μM and by 63.49 and 108.77%, respectively, at 3000 μM (Figure 6). CV significantly inhibited PDB-induced contraction only at 3000 μM, resulting in a total (101.69%) blockade. 

## 3. Discussion

The present study compared for the first time the effect of POH, CV, and LM on VSM in the rat aorta. Our main finding indicates that POH, CV, and LM had a relaxant effect on several types of contraction. Moreover, in spite of a similar molecular structure, substances with a –OH group had relative specificity for blocking the EMC.

We here investigated the effect of POH, CV, and LM on two types of excitation-contraction coupling: EMC and PMC. In terms of EMC, all substances had a relaxant effect in K^+^-induced contraction. In EMC, intracellular Ca^2+^ concentration ([Ca^2+^]_i_) increases after direct activation of voltage-operated Ca^2+^ channels (VOCCs) following membrane depolarization, thus leading to contraction [34]. POH and CV relaxed the tonus of aortic ring preparations, which was artificially increased by adding 60 mM [K^+^] to the bathing solution, promoting cytoplasmic membrane depolarization to values close to −15 mV and outward K^+^ rectification [35]. This depolarization shows that the effect elicited by POH and CV is independent of primary trans-membrane electric potential alteration. At this transmembrane potential, VOCCs are activated, increasing Ca^2+^ inflow to the cytoplasm and triggering EMC. Given that VOCC activation is essential for vasoconstriction in EMC, we hypothesized that inhibition of K^+^-induced contraction by POH and CV was caused by direct VOCC blockade. To corroborate this point, POH and CV showed smaller EC_50_ values in EMC than in PMC (Table 1). We therefore tested whether both monoterpenoids blocked contraction induced by BayK-8644, an agonist of L-type VOCCs [36]. Consistent with our hypothesis, POH and CV inhibited BayK-8644 contraction. Furthermore, inhibition of BayK-8644-induced contraction occurred with similar pharmacological potency and efficacy as during blockade of K^+^-induced contraction (Table 1), indicating that POH and CV inhibit K^+^-induced contraction likely to blocking L-type VOCC.

Comparison of EC_50_ values and pharmacological efficacy on blocking EMC in preparations with and without endothelium, showed no difference between CV and POH. This indicates that their effects on EMC during VSM contraction are not modulated by endothelial factors.

Similarly to our results, previous studies suggested that some terpenes and terpenoids, such as terpinen-4-ol, thymol, carvacrol, eugenol, and *trans*-caryophyllene, relaxed smooth muscles via VOCC blockade [9,10,37,38]. This provides further support to our hypothesis that CV and POH relaxation of VSM preparations involves blocking VOCCs in the plasma membrane.

Concerning PMC, all substances promoted relaxation of VSM in this type of contraction. An increase in [Ca^2+^]_i_ is necessary for VSM contraction; it can be triggered by agonist-induced activation of membrane receptors which, in turn, activate receptor-operated Ca^2+^ channels (ROCC) [39]. In this study, we used PHE and PDB as agonists to activate PMC. PHE is an α1-adrenoceptor agonist, which induces Ca^2+^ influx through ROCC, promotes Ca^2+^ release from the sarcoplasmic reticulum, and results in Ca^2+^ sensitization of contractile proteins [40]. α1-adrenoceptor activation initially elicits a phasic contraction component, with Ca^2+^ release from the sarcoplasmic reticulum promoted by interaction between inositol trisphosphate (IP_3_) and IP_3_ receptors, followed by a sustained tonic phase [40]. In the tonic phase, contraction is sustained by Ca^2+^ influx through ROCC and Ca^2+^-independent pathways. These pathways involve contractile machinery sensitization by biochemical intracellular substance like PKC [41]. The full blockade of PHE-induced contraction shows that POH and CV could block all components of this contraction, suggesting that, although with smaller pharmacological potency, there are other mechanisms of action involved besides VOCC blockade. Additionally, comparison of POH and CV EC_50_ values for blocking PHE-stimulated PMC, shows no modulation by the endothelium, as already observed during EMC.

Our data on the effect of CV and POH on PHE-induced contraction differ from previously reported findings, whereby other terpenoids’ pharmacological effect was modulated by the endothelium [10,14,42]. This is important because in some diseases such as arterial hypertension, the endothelium is damaged and its influence is lost [43]. Moreover, it shows the richness and variety of pharmacological effects of these natural terpenoids.

LM was the only substance without a –OH group in its molecule. Its activity on K^+^- and PHE-induced contractions showed a strikingly different pharmacological activity profile, compared to POH and CV. In general, its pharmacological potency was smaller and was partially dependent on endothelium preservation. In endothelium-denuded preparations, the pharmacological potency of LM in blocking K^+^- and PHE-induced contractions varied significantly and was much greater during PMC. The opposite occurred during EMC, whereby preparations with preserved endothelium showed increased pharmacological potency. Accordingly, the absence of –OH not only decreased the potency, but also the relative specificity of POH and CV in causing this effect. The impact of the endothelium on LM treatment was not surprising, as the endothelium releases several factors that modify the intensity of muscle contraction [44,45]. It should be noted that the alcohols POH and CV are more potent than non-alcoholic LM and a comparison of EC_50_ values on endothelium-denuded preparations showed the order of pharmacological efficacy to be: LM < CV < POH.

BayK-8644 induces contraction by L-type VOCC activation [36]. Assuming that inhibition of BayK-8644-induced contraction was due to blockade of VOCCs, then the EC_50_ values for blocking of BayK-8644- and 60 mM K^+^-induced contractions were expected to be similar. This was indeed the case for POH and CV, but not for LM. Nevertheless, the result for LM does not invalidate the assumption of VOCC blockade in the case of POH and CV. The antagonistic effect of a given agent might rely on different mechanisms. For example, nifedipine and verapamil block BayK-8644 contraction via competitive and non-competitive mechanisms, respectively [46], and this could be the case also for LM. Although we do not know the mechanism of LM blockade for BayK-8644-induced contraction, it is reasonable to suggest that it differs from that of POH and CV. This would explain why LM could only partially (< 80%) block BayK-8644-induced contractions.

POH, CV, and LM could inhibit the contraction induced by PDB, a phorbol ester. PDB acts primarily as a PKC activator and causes Ca^2+^ sensitization of the contractile machinery, eliciting contraction without substantial changes in myoplasmic Ca^2+^ concentration [32,47]. In the present study, PDB used a mechanism of action that did not involve Ca^2+^ inflow to the cell, as this set of experiments was performed in Ca^2+^-free solution. POH, CV, and LM acted with grossely similar pharmacological potency on PDB-induced contraction (Figure 6). They all showed greater potency in blocking EMC than on PDB contraction. This fact reinforces the suggestion that the three substances employ several mechanisms of action and that presence of –OH on the molecule increases the potency on EMC, as shown also by EC_50_ values.

This effect shows that LM may induces mechanisms that triggers contraction, for example, release calcium from sarcoplasmatic reticulum like other monoterpenes [9]. Furthermore, in endothelium-intact preparations, but not in endothelium-denuded, LM showed a potentiating effect in PMC at 600 μM. Taken together, these results suggest that endothelium participate on contraction effects induced by LM, perhaps by means of release of contraction-inducing factors, such as, prostaglandins [48,49], endothelin [50,51] or other unknown factor.

The three monoterpenes in this study were able to relax contractions elicited by both EMC and PMC in a concentration-dependent manner. However, their potencies differed with respect to presence and position of the –OH group. Comparison of EC_50_ values in endothelium-denuded preparations revealed this order of pharmacological potency: LM < CV < POH. The hydroxylated molecules, CV and POH, exhibited greater relaxant effects than non-hydroxylated LM. Among the hydroxylated compounds, POH, a primary alcohol (i.e., –OH is attached to a primary carbon atom) and very hydrophilic, is more potent than CV, a secondary alcohol. Thus, the observed order of pharmacological potency reflects the degree of hydrophilicity imparted by the presence and position of the –OH group. Several studies support our hypothesis that –OH is important for the pharmacological and biological activity of these compounds [20,52].

Interestingly, some studies demonstrated the importance of the –OH group in monoterpenoids for their action on ion channels. During TRPV3 activation, for example, the six most potent monoterpenes carried a hydroxyl group, whereas none of the compounds without an oxygen moiety (p-cymene and (+)-limonene) affected the channel to a significant extent [22]. These studies strengthen our hypothesis that CV and POH may act on muscle cells by affecting ion channels such as VOCCs.

## 4. Materials and Methods

### 4.1. Animals

Male Wistar rats, weighing 200–300 g, were kept under conditions of constant temperature (23 ± 2 °C) with a 12 h light/12 h dark cycle and free access to food and water. All animals were handled in compliance with the Guide for the Care and Use of Laboratory Animals, 8th edition, published by the US National Institutes of Health in 2011 (https://www.nap.edu/read/12910/chapter/1) and all efforts were made to minimize animal suffering. All procedures were approved by the Animal Care and Use Committee of the State University of Ceará (process approval number 12237313-0).

### 4.2. Solutions and Drugs

Krebs Henseleit’s solution (KHS) was used as bathing solution with the following composition: 118 mM NaCl, 4.7 mM KCl, 25 mM NaHCO_3_, 2.5 mM CaCl_2_·2H_2_O, 1.2 mM KH_2_PO_4_, 1.2 mM MgSO_4_·7H_2_O, 11 mM glucose, and 0.01 mM EDTA. The KHS was constantly aerated (5% O_2_, 95% CO_2_) and the pH was adjusted to 7.40 with NaOH/HCl. In experiments requiring Ca^2+^-free solutions, equimolar substitution of CaCl_2_ with EGTA (2 mM) was carried out. The agonists PHE and acetylcholine (Ach) were first dissolved in distilled water to make stock solutions. LM, CV, and POH were first diluted in Tween 80 (final concentration ≤ 0.05%). BayK-8644, PDB, and thapsigargin were first diluted in dimethyl sulfoxide (final concentration ≤ 0.05%). All stock solutions were sonicated immediately before addition to the experimental chamber to attain the final desired concentration. All reagents were of analytical grade and were purchased from Sigma Chemical Co. (St. Louis, MO, USA), Reagen (Rio de Janeiro, RJ, Brazil), or Vetec (Rio de Janeiro, RJ, Brazil).

### 4.3. Isometric Tension Recording

Tissue preparation and isometric tension recordings were made as previously reported [10]. Briefly, rats were sacrificed by CO_2_ inhalation followed by exsanguination. The thoracic aorta was dissected and connective tissue was removed in KHS. Afterwards, the aorta was cut transversally into cylindrical segments of approximately 4 mm in length and these were mounted on an isolated organ chamber containing KHS gassed with (95% O_2_/5% CO_2_), at 37 °C. Mechanical activity was recorded by an isometric force transducer (FTO3; Grass Instruments Co., Quincy, MA, USA) connected to an acquisition system (PM-1000; CWE Inc., Akron, OH, USA). Aortic rings were stretched with a passive tension of 1 g and allowed to equilibrate for 60 min. Vessel contractility was then tested by an initial exposure to a high-[K^+^] (60 mM) solution. Experiments were performed in the presence or absence of endothelium. When required, the endothelium was denuded by gentle rubbing of the aortic lumen with a stainless steel wire. Successful endothelium removal was confirmed by the absence of relaxation of arteries pre-contracted with PHE (0.1 μM) and then exposed to Ach (1.0 μM) to induce more than 70% relaxation.

After assessing tissue viability and the endothelium, the following experiments were performed using the compounds in paired form. For a given experiment, a recording channel was assigned to each substance and one channel solely to the external control. LM, CV, or POH were cumulatively added to the organ chamber to allow for increasing concentrations to be achieved (1–5000 μM) and their effect on the resting tone of endothelium-containing aortic rings was evaluated. In a subset of experiments, concentration-dependent responses to LM, CV, or POH were measured in endothelium-containing and endothelium-denuded rings pre-contracted with either 60 mM KCl or 0.1 μM PHE. In another series of experiments, the effects of LM, CV, and POH were compared against the contractions induced by PDB (1 μM), a PKC activator [32], in endothelium-denuded rings incubated in Ca^2+^-free KHS (added 2 mM EGTA) and previous (30 min) addition of thapsigargin (1 μM), a calcium pump inhibitor [33]. Relaxant effects of increasing concentrations of LM, CV, and POH on the sustained contractile response to BayK-8644 (2 μM) were studied in endothelium-denuded aortic rings that had been sensitized with extracellular [K^+^] of regular KHS increased to 10 mM. Control experiments were made in the sole presence of the vehicle (Tween 80) at the same concentrations used to dilute the monoterpenes.

### 4.4. Statistical Analysis

Data are expressed as mean ± standard error of the mean (SEM), where (*n*) indicates the number of experiments. Concentration-response curve and EC_50_ values for LM, CV, and POH were made and adjusted with the Hill equation as follows:

$$\%\;\mathrm{of}\;\mathrm{agonist}\;\mathrm{contraction}=\min+\frac{\max-\;\min}{1+{\left(\frac{\left[\mathrm{agonist}\right]}{{\mathrm{EC}}_{50\%}}\right)}^{k}}$$

where min and max are the minimum and maximum contraction force values, [agonist] is the agonist concentration, and *k* is the Hill slope. The significance for statistical tests was *p* < 0.05 and to compare data from different groups, one- or two-way ANOVA was used, followed by Holm-Sidak’s test for parametric data and ANOVA on Rank’s followed by Dunn’s test for non-parametric data.

## 5. Conclusions

In conclusion, the results show that limonene, carveol and perillyl alcohol have an important relaxant effect on the contractility of vascular smooth muscle cells. POH and CV had higher pharmacological potency on the electromechanical coupling, suggesting that a blockade of Ca^2+^ channels are likely to contribute to the monoterpene-induced vasorelaxation, although we cannot exclude other mechanisms of action, such as, for example, inhibition of PKC and IP_3_ pathway. Although not crucial for monoterpene bioactivity, the hydroxyl group is relevant for the pharmacological potency of these compounds.

## Figures and Tables

**Table 1:** EC_50_ values summarizing the relaxant effects of perillyl alcohol, carveol, and limonene on rat aortic rings. Values are expressed as means ± SEM.

Contractile Agent		Perillyl Alcohol (µM)	Carveol (µM)	Limonene (µM)
K^+^	E+	277.7 ± 5.46 (6)	662.1 ± 32.85 (7) ^a^	941.6 ± 28.02 (6) ^b^
K^+^	E−	279.7 ± 22.01 (7)	619.8 ± 37.15 (7) ^a^	1474.5 ± 27.08 (7) ^b,c^
PHE	E+	443.3 ± 66.83 (6) ^d^	1333.3 ± 225.20 (10) ^a,e^	2159.1 ± 203.62 (7) ^b,f^
PHE	E−	433.3 ± 44.31 (7) ^d^	1237.34 ± 117.90 (9) ^a,e^	1216.7 ± 57.50 (7) ^c,f^
BayK	E−	221.4 ± 4.09 (5)	598.2 ± 42.05 (5) ^a^	439.0 ± 31.76 (5) ^b,g^

E+, endothelium-intact aorta; E−, endothelium-denuded aorta; PHE, phenylephrine; ^a^, *p* < 0.05, in compared to the corresponding POH value; ^b^, *p* < 0.05, compared to the corresponding CV value; ^c^, *p* < 0.05, endothelium-intact vs. endothelium-denuded; ^d^, *p* < 0.05, compared to 277.7, 279.7, and 221.4 µM; ^e^, *p* < 0.05 compared to 662.1, 619.8, and 598.2 µM; ^f^, *p* < 0.05, compared to 941.6, 1474.5, and 439.0 µM; ^g^, *p* < 0.05, BayK vs. limonene K^+^ E+ and K^+^ E−; Values are expressed as means ± SEM (*n*), *n* = number of experiments.
